# Blood Cell DNA Methylation of Aging-Related Ubiquitination Gene *DZIP3* Can Predict the Onset of Early Stage Colorectal Cancer

**DOI:** 10.3389/fonc.2020.544330

**Published:** 2020-11-27

**Authors:** Yuan Quan, Fengji Liang, Deqing Wu, Xueqing Yao, Zhihuang Hu, Yuexing Zhu, Ying Chen, Andong Wu, Danian Tang, Bingyang Huang, Ruifeng Xu, Zejian Lyu, Qian Yan, Lang Luo, Zhengzhi Ning, Yong Li, Jianghui Xiong

**Affiliations:** ^1^School of Computer Science and Technology, Harbin Institute of Technology Shenzhen Graduate School, Shenzhen, China; ^2^Lab of Epigenetics and Advanced Health Technology, Space Science and Technology Institute (Shenzhen), Shenzhen, China; ^3^State Key Laboratory of Space Medicine Fundamentals and Application, China Astronaut Research and Training Center, Beijing, China; ^4^Department of General Surgery, Guangdong Provincial People’s Hospital, Guangdong Academy of Medical Sciences, Guangzhou, China; ^5^Department of Medical Oncology, Fudan University Shanghai Cancer Center, Shanghai, China; ^6^Gastro-Intestinal Surgery Department, Beijing Hospital, Beijing, China; ^7^Department of Cardiothoracic Surgery, Strategic Support Force Medical Center of PLA. No. 9, Beijing, China; ^8^Shenzhen Taikontek Health Technology Co., Ltd, Shenzhen, China

**Keywords:** colon cancer, aging immune system, E3 ubiquitin ligase, liquid biopsy, cancer screening, early detection, *DZIP3*

## Abstract

There is a body of evidence that the aging immune system is linked to cancer. In this study, with aging- and immune-related DNA methylation data, we investigated the DNA methylation regulation changes in promoters with other regions of genes during aging and their association with the immune-cell proportion in the circulating whole blood of individuals. The analyses for aging- and CD4+ T cell proportion-derived differential genes showed that ubiquitination plays an important role in the aging immune system and tumorigenesis. Therefore, starting from a set of pre-annotated ubiquitination genes, we found that among the differentially ubiquitinated genes, *DZIP3*, an E3 ubiquitin ligase with no reports on its function in immune cells and tumorigenesis, was significantly associated with both aging (P-value = 3.86e-06) and CD4+ T cell proportion (P-value = 1.97e-05) in circulating blood. By collecting a cohort of 100 colon cancer patients and 50 healthy individuals, we validated that the 1^st^ exon DNA methylation of *DZIP3* could predict the onset of early stage (AUC = 0.833, OR = 8.82) and all pTNM stages of colorectal cancer (AUC = 0.782, OR = 5.70). Thus, the epigenetically regulated ubiquitination machine plays an important role in immune aging and tumorigenesis.

## Introduction

In recent years, colorectal cancer (CRC) has gradually developed into the third most common cancer worldwide and is one of the leading causes of cancer-triggered deaths (1). In the past few decades, due to a large number of studies on the pathogenesis of CRC, the diagnostic tests and treatment strategies have made significant progress, which has led to the improvement of CRC survival time (2).

Previous studies have found that there are several pathogenic factors of cancer, such as genome instability and mutation, intestinal flora imbalance and unreasonable diet (3). Immune destruction is also an important cause of cancer. Therefore, chronic inflammation is one of the hallmarks of cancer (3). It is well known that the earliest stages of tumorigenesis are often accompanied by the appearance of chronic inflammation (4), and CRC is considered to be one of the most representative examples of tumors closely related to chronic inflammation (5). A large number of studies have shown that prolonged inflammation of the intestine, such as Crohn’s disease or ulcerative colitis and other inflammatory bowel diseases (IBDs), often develop into CRC at later stage (6–8). Notably, studies have found that individuals can prevent or delay CRC by using anti-inflammatory drugs (9, 10), which also suggests that the inflammatory process is closely linked to tumor onset. The inflammatory reactions are usually associated with microbial responses, and the intestinal tract is composed of numerous bacterial strains. Under normal circumstances, these bacterial strains in harmony with their human hosts. However, any substantial change in the bacterial population may have a significant impact on inflammatory responses and lead to tumorigenesis.

In addition, age is a major risk factor for many cancers. There is a body of evidence that aging immune system is linked to cancer (11–13). There is a growing interest in immunosenescence and how it may contribute to the increased risk of cancer onset with aging. For example, CD4+ T cells, especially T follicular helper cells, are essential for the body to produce the robust humoral response against infection and vaccination (14). Evidence suggested that the aging microenvironment can cause age-related functional deficits in mouse CD4+ T cells (15). And age-related damage of the humoral response to influenza is related to disturbances of antigen-specific T follicular helper cell responses (16).

On the other hand, ubiquitination refers to the covalent attachment of ubiquitin molecules with proteins, which is a mechanism widely used by organisms to rapidly regulate cell signaling. Recent studies showed that ubiquitination has an important role in regulating various signals of innate and adaptive immune cells (17–19). A good example is that PD-1 ubiquitination could regulate the antitumor immunity of T cells. In the tumor microenvironment, PD-1 of dysfunctional T cells is abnormally highly expressed; hence antibody inhibitors against PD-1 or its ligand (PD-L1) have become one of the commonly used drugs for the treatment of various tumors. A study demonstrated that the surface PD-1 protein of activated T cells undergoes internalization, followed by ubiquitination and proteasome degradation (20). Here, we hypothesized that ubiquitination might play a key gatekeeper role in immune system aging and tumorigenesis in CRC.

The study of tumor immune interactions presents great technical challenges due to the great heterogeneity in the tumor microenvironment and global immune system function. Blood cell DNA methylome analysis provides a promising tool to probe the key components of immune cell dysregulation in aging and tumorigenesis. In recent years, researchers have identified several DNA methylation markers for quantification of tumor immune cell infiltration, which have significantly improved specificity compared to other markers. For instance, FOXP3’s DNA methylation value can be used as a marker to distinguish T lymphocyte subpopulations, which can distinguish regulatory T lymphocytes from other types of T lymphocytes (21). However, because these T lymphocyte subpopulations can express similar surface markers, it is difficult for researchers to distinguish based on other common methods, such as gene expression or immunohistochemistry (21, 22). With the development of DNA methylation signature, that can coinstantaneous quantification of diverse immune cell populations in human blood, the ability of DNA methylation as a cell typing tool will be further enhanced. There are reports that a broad signature of hepatocellular carcinoma (HCC) in peripheral blood mononuclear cell and T cell DNA methylation, which can distinguish HCC from chronic hepatitis B and C as well as healthy individuals in the early stage, escalates with the progression of HCC (23).

In the present study, we will systematically study the DNA methylation of ubiquitination regulatory system genes in blood cells and screen candidate genes that are correlated with both aging and immune cell compositions. Then, we will use low-cost nucleic acid mass spectrometry (Sequenom) to test whether ubiquitination genes can be used as candidate markers for the early screening of CRC.

## Materials and Methods

### Identification of Differential Genes Associated With Aging and Immune Cell Proportions

In our study, aging-related and immune-related DNA methylation data were collected from the Gene Expression Omnibus (GEO) database (www.ncbi.nlm.nih.gov/geo, GEO accession number: GSE40279 and GSE69270). GEO is an open-source functional genomics database composed of high-throughput resources, including microarray data, gene expression data, and DNA methylation data, *etc*. The DNA methylation platform’s annotation information was used to transform the probes into homologous gene symbols. The GSE40279 dataset contained DNA methylation profiles across approximately 450K CpGs in the human whole blood of 656 samples from patients whose age ranged from 19 to 101 years. The GSE69270 dataset contained DNA methylation profiles in the whole blood of 184 samples with annotations of six immune cell proportions, including CD8+ T and CD4+ T cells, monocytes, granulocytes, and NK and B cells. Next, we calculated the significantly differential genes during aging and their association with immune cell proportion in the circulating whole blood of healthy individuals.

**Figure 1:**
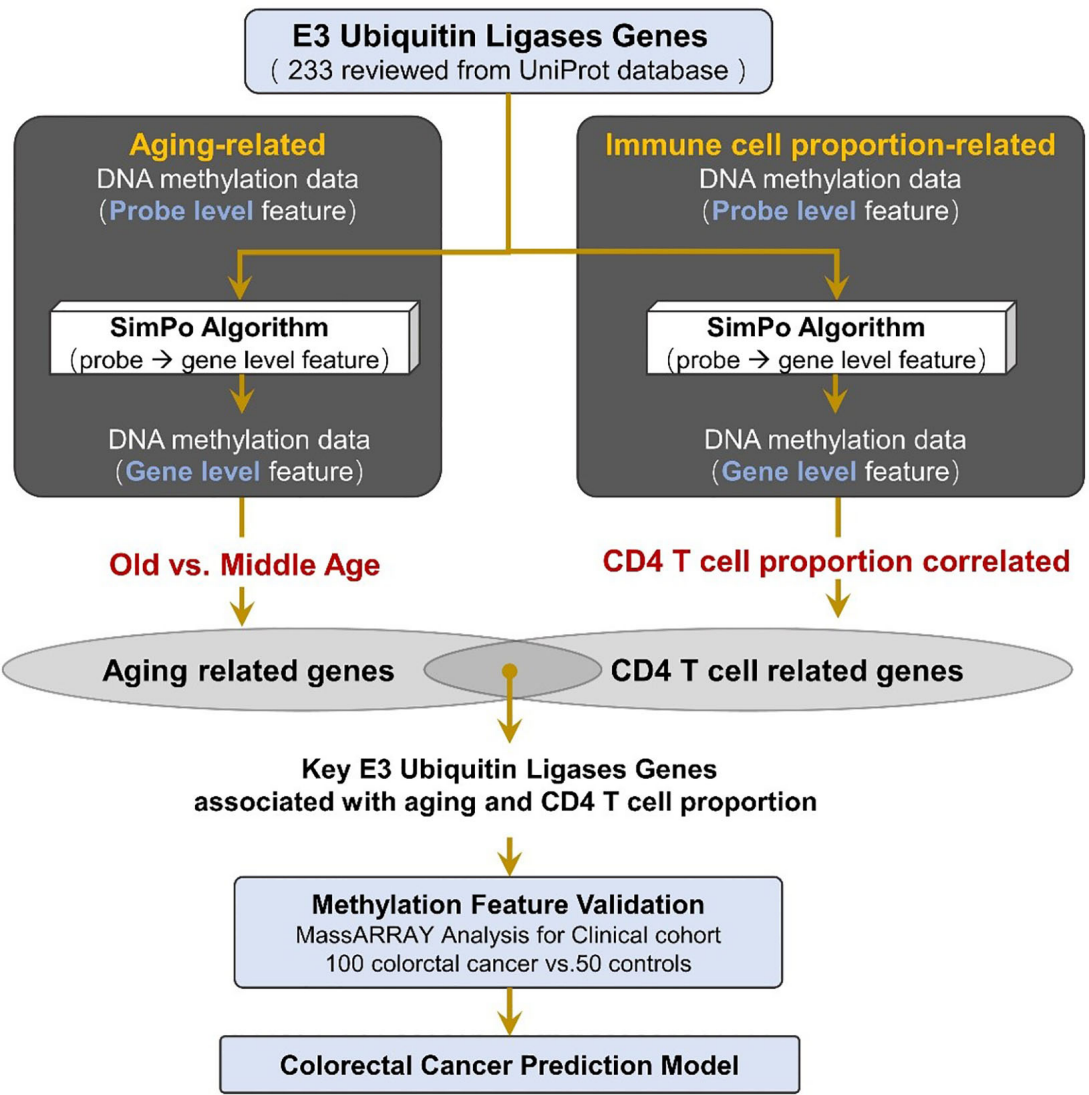
Method pipeline of candidate marker validation and prediction model construction for colorectal cancer.

Recently, a study published in *Genome Res*. found that there is a strong relationship between the methylation of gene body difference to promoter (MeGDP) and gene expression, which correlation coefficient is up to 0.67 (24). Accordingly, MeGDP can be used as a predictor of gene expression. The higher the MeGDP is, the higher the expression value of a gene, suggesting that MeGDP is closely linked to the corresponding phenotypes. Depending on the results of the above research, our other paper proposed the Statistical Difference of DNA Methylation between Promoter and other regions (SIMPO) to calculate the DNA methylation characteristics of different genes (**Figure 1**) (25). Recently, based on SIMPO algorithm, our group has achieved promising results in the research of DNA methylation biomarker identification for type 2 diabetes (26).

The input data of the SIMPO algorithm is the DNA methylation values of cg probes that are located in the gene promoter and the other regions. The significant difference method T-test is used in the SIMPO algorithm, and the degree of difference (SIMPO score) is used to characterize the DNA methylation of corresponding genes as follows (21):

$$SimPo\;score=\frac{\bar{x}-\bar{y}}{\sqrt[{S_{w}}]{\frac{1}{m}+\frac{1}{n}}}∼t(m+n-2)$$

where

$$S_{w}^{2}=\frac{1}{m+n-2}[(m-1)S_{1}^{2}+(n-1)S_{2}^{2}]$$

$\bar{x}$: average DNA methylation value of all probes that are located in the promoter region; $\bar{y}$: average DNA methylation value of all probes that are located in the other region; m: number of probes that are located in the promoter region; n: number of probes that are located in the other region;$S_{1}^{2}$ : variance of DNA methylation values of probes that are located in the promoter region; and $S_{2}^{2}$: variance of DNA methylation values of probes that are located in the other region. In addition, since the SIMPO score relates to the number of probes, to ensure the reliability of the SIMPO score, we only selected genes with a number of promoter region-located and other region-located probes greater than or equal to five for further calculation.

Based on the SIMPO algorithm, this study separately calculated the SIMPO score of each gene in the aging-related and immune cell proportion-related DNA methylation data. Next, we calculated the differences of gene SIMPO scores between the old age (>50 year) and middle age (≤50 year) individuals based on the adjusted T-test. Then, for the immune-related methylation data, we identified the differential genes that were significantly associated with immune cell proportion based on the adjusted Spearman correlation test.

### Ubiquitination Genes Collection

In this study, we collected 233 reviewed E3 ubiquitin ligase genes in humans by querying the UniProt database (https://www.uniprot.org) with “E3 ubiquitin-protein ligase” as a key word (**Figure 1**, **Table S1**).

The biological functions were enriched for 233 E3 ubiquitin ligases using the Kyoto Encyclopedia of Genes and Genomes (KEGG) pathway records in the Database for Annotation, Visualization and Integrated Discovery (DAVID, https://david.ncifcrf.gov/) (27). The corresponding catalogs and diseases of the KEGG pathways were downloaded from the KEGG Pathway Database (https://www.genome.jp/kegg/pathway.html) (28). The results showed that these E3 ubiquitin ligases were enriched in four KEGG pathways (Bonferroni-adjusted P-value ≤ 0.05), of which the most significant is the ubiquitin-mediated proteolysis pathway (**Table S2**). This result indicates that these 233 E3 ubiquitin ligases are significantly associated with ubiquitination and could be used for the next analysis.

### Survival Analysis

In this study, we performed a Kaplan–Meier survival analysis and a multivariate Cox regression analysis to examine the relationship between the DNA methylation level (SIMPO scores) of E3 ubiquitin ligase genes and survival times of colon adenocarcinoma (COAD) patients. The methylation data of the COAD patient’s tumor tissue and their clinical survival information were downloaded from the Cancer Genome Atlas (TCGA, https://portal.gdc.cancer.gov), which contain 293 individuals. According to the values of SIMPO scores, the patients with COAD were divided into high-expression (top 50%) and low-expression (bottom 50%) groups for each gene. Then, SangerBox package (http://sangerbox.com/) was utilized to plot Kaplan–Meier curves for SIMPO scores, and the significances of the difference between Kaplan–Meier curves of these two groups were calculated separately for each gene by the Log-rank tests. In addition, the overall survival analysis also contained the Cox proportional hazard ratio (HR) and the 95% confidence interval. Finally, P-value <0.05 was considered to indicate the statistically significant result.

### Clinical Blood Samples Collection

We analyzed blood samples from 100 diagnosed CRC patients (male: 67, female: 33) from Guangdong Provincial People’s Hospital and FoShan New RongQi Hospital. A total of 53% of the samples were diagnosed with pTNM stage I/II. We also collected 50 normal control samples (male: 18; female: 32) from FoShan New RongQi Hospital. All peripheral whole blood cell samples were treated with EDTA anticoagulant.

### MassARRAY Analysis

We identified the sequences 500 bp up and down from the CpG position at the 1st exon of *DZIP3* (probe cg14787155 annotated position in HM450K chip) with the UCSC genome browser (http://genome.ucsc.edu/) (chr3:108308033-108309033), and designed one primer set for methylation analysis of the ***amplicon-cg14787155*** region by EpiDesigner software (http://epidesigner.com). Quantitative DNA methylation analysis of ***amplicon-cg14787155*** was carried out by using the MassARRAY platform (SEQUENOM) according to the official pipelines. In brief, DNA was treated with sodium bisulfite and PCR amplified, and bisulfite reactions were designed. The DNA methylation status of the samples was quantitatively tested by using the matrix-assisted laser desorption ionization time-of-flight mass spectrometry. Finally, 25 CpG sites were tested, and the methylation data of 18 individual units (one to two CpG sites per unit) were generated by the EpiTyper v1.0.5 software.

## Result

### Biological Analysis of Aging and CD4+ T Cell Proportion-Derived Differential Genes

Based on the SIMPO algorithm, we calculated the SIMPO score for each gene of the aging and immune-related DNA methylation data. In the aging-related methylation data, we identified a total of 2,358 genes that were significantly associated with the age of the patient (based on the adjusted T-test between the old age (>50 year) and middle age (≤50 year) individuals). In the immune-related methylation data, we identified a total of 1,331 genes that were significantly associated with the CD4+ T cell proportion (based on the adjusted Spearman correlation test).

First, we performed the KEGG pathway enrichment analyses for the above significant gene sets by using the Enrichr database (https://amp.pharm.mssm.edu/Enrichr/) (29). The results showed that the aging-associated genes were enriched in 32 significant KEGG pathways, and the immune-associated genes were enriched in 23 significant KEGG pathways. Among them, it is worth noting that the cellular senescence pathway is enriched in both the aging- and immune-associated gene sets (**Table 1**). In addition, the ubiquitin-mediated proteolysis pathway was identified by an immune-related gene set (**Table 1**). Therefore, the results indicated that genes that are significantly disturbed in DNA methylation level during the aging and immunization processes are associated with ubiquitination.

**Table 1:** The significantly enriched KEGG pathways of the aging- and CD4+ T cell proportion-derived differential genes (top 20).

Aging		CD4+ T cell proportion	
KEGG pathway	P-value	KEGG pathway	P-value
Glyoxylate and dicarboxylate metabolism	7.83e-05	Ferroptosis	1.04e-03
Non-small cell lung cancer	4.67e-04	Cysteine and methionine metabolism	3.40e-03
Axon guidance	1.11e-03	Platelet activation	8.16e-03
Cell cycle	2.39e-03	Cellular senescence	1.01e-02
Cellular senescence	6.67e-03	Non-small cell lung cancer	1.15e-02
Nucleotide excision repair	7.05e-03	AMPK signaling pathway	1.35e-02
DNA replication	7.18e-03	TNF signaling pathway	1.44e-02
Valine, leucine and isoleucine degradation	8.41e-03	Hepatitis C	1.50e-02
Melanoma	8.68e-03	Hippo signaling pathway	2.01e-02
Human cytomegalovirus infection	8.70e-03	Base excision repair	2.02e-02
C-type lectin receptor signaling pathway	9.24e-03	Fatty acid degradation	2.51e-02
Dopaminergic synapse	1.00e-02	Viral carcinogenesis	2.70e-02
Oocyte meiosis	1.07e-02	Chronic myeloid leukemia	2.87e-02
Glioma	1.29e-02	Cushing syndrome	2.91e-02
Pancreatic cancer	1.29e-02	Tyrosine metabolism	3.00e-02
Hepatitis C	1.40e-02	Fatty acid elongation	3.07e-02
Chronic myeloid leukemia	1.46e-02	Progesterone-mediated oocyte maturation	3.14e-02
TNF signaling pathway	1.72e-02	Glycosaminoglycan degradation	3.39e-02
Ubiquitin mediated proteolysis	1.72e-02	Lysosome	3.42e-02
Hippo signaling pathway	2.08e-02	Leukocyte transendothelial migration	3.51e-02

In addition, the Gene Ontology (GO) molecular function results further validate the above conclusion. The aging-derived differential genes were enriched in the ubiquitin-like protein ligase binding and ubiquitin protein ligase binding terms (**Table 2**). CD4+ T cell proportion-derived differential genes were also enriched in the ubiquitin binding term (**Table 2**).

**Table 2:** The significantly enriched GO molecular function terms of the aging- and CD4+ T cell proportion-derived differential genes (top 20).

Aging		CD4+ T cell proportion	
GO term (molecular function)	P-value	GO term (molecular function)	P-value
RNA binding	8.10e-07	Microtubule plus-end binding	9.81e-05
Protein-DNA loading atpase activity	3.88e-04	Kinase binding	5.82e-04
DNA clamp loader activity	3.88e-04	Phosphatidylinositol 3-kinase regulator activity	1.10e-03
Bubble DNA binding	3.88e-04	RNA binding	1.70e-03
Microtubule plus-end binding	2.19e-03	Protein kinase binding	2.23e-03
Ubiquitin-like protein ligase binding	3.96e-03	Clathrin adaptor activity	2.96e-03
Ubiquitin protein ligase binding	4.26e-03	Endocytic adaptor activity	2.96e-03
5′-3′ RNA polymerase activity	7.76e-03	Prenyltransferase activity	4.41e-03
Aminoacyl-trna ligase activity	7.93e-03	Transcription corepressor activity	4.74e-03
3-hydroxyacyl-coa dehydrogenase activity (GO:0003857)	9.11e-03	Neurotrophin TRKA receptor binding	5.05e-03
Kinase activity (GO:0016301)	1.32e-02	RNA polymerase II transcription factor activity, sequence-specific transcription regulatory region DNA binding	6.86e-03
Protein kinase binding	1.40e-02	Ubiquitin binding	8.33e-03
Signal sequence binding	1.55e-02	Ribosomal protein S6 kinase activity	8.40e-03
Acetyltransferase activity	2.21e-02	Protein-DNA loading atpase activity	8.40e-03
Clathrin adaptor activity	2.25e-02	DNA clamp loader activity	8.40e-03
G-rich strand telomeric DNA binding	2.25e-02	Single-stranded DNA endodeoxyribonuclease activity	8.40e-03
I-SMAD binding	2.25e-02	N-methyltransferase activity	1.08e-02
Magnesium ion transmembrane transporter activity	2.25e-02	Hydrolase activity, hydrolyzing N-glycosyl compounds	1.14e-02
Endocytic adaptor activity	2.25e-02	Ligand-dependent nuclear receptor transcription coactivator activity	1.15e-02
Double-stranded DNA binding	2.37e-02	Damaged DNA binding	1.16e-02

The GO biological process results show that the aging-derived differential genes were enriched in the proteasome-mediated ubiquitin-dependent protein catabolic process and ubiquitin-dependent protein catabolic process terms (**Table 3**). The CD4+ T cell proportion-derived differential genes were enriched in the positive regulation of the proteasomal ubiquitin-dependent protein catabolic process, the regulation of the proteasomal ubiquitin-dependent protein catabolic process and protein polyubiquitination (**Table 3**).

**Table 3:** The significantly enriched GO biological process terms of the aging- and CD4+ T cell proportion-derived differential genes (top 20).

Aging		CD4+ T cell proportion	
GO term (biological process)	P-value	GO term (biological process)	P-value
DNA metabolic process	3.23e-05	Positive regulation of proteasomal ubiquitin-dependent protein catabolic process	5.46e-05
Cellular macromolecule biosynthetic process	3.34e-05	Positive regulation of proteasomal protein catabolic process	3.28e-04
Translation	3.71e-05	Cellular response to DNA damage stimulus	3.55e-04
Regulation of histone modification	5.74e-05	Negative regulation of protein serine/threonine kinase activity	4.77e-04
Cellular response to DNA damage stimulus	7.21e-05	Retinoic acid receptor signaling pathway	5.81e-04
Transcription, DNA-templated	7.45e-05	Regulation of proteasomal ubiquitin-dependent protein catabolic process	7.40e-04
Transcription-coupled nucleotide-excision repair	8.76e-05	Pyruvate metabolic process	8.84e-04
DNA repair	1.18e-04	Positive regulation of smooth muscle cell apoptotic process	1.10e-03
Nucleotide-excision repair	1.25e-04	Negative regulation of response to biotic stimulus	1.48e-03
Proteasome-mediated ubiquitin-dependent protein catabolic process	1.56e-04	Regulation of transcription from RNA polymerase ii promoter	1.61e-03
Mitochondrial gene expression	1.62e-04	DNA repair	1.73e-03
Proteasomal protein catabolic process	2.54e-04	Negative regulation of protein phosphorylation	1.84e-03
RNA processing	2.84e-04	Toll-like receptor 3 signaling pathway	1.88e-03
Positive regulation of DNA biosynthetic process	5.41e-04	Negative regulation of cyclin-dependent protein kinase activity	1.96e-03
Gene expression	6.40e-04	Negative regulation of peptidyl-threonine phosphorylation	2.21e-03
TRNA aminoacylation for protein translation	7.50e-04	Regulation of defense response to virus by virus	2.43e-03
Positive regulation of chromatin silencing	9.33e-04	Protein polyubiquitination	2.49e-03
Regulation of endodeoxyribonuclease activity	9.33e-04	Hexose biosynthetic process	2.50e-03
Postreplication repair	9.45e-04	Response to laminar fluid shear stress	2.96e-03
Ubiquitin-dependent protein catabolic process	1.03e-03	G1 DNA damage checkpoint	2.96e-03

Furthermore, we performed the disease association analysis of the aging- and immune-related gene sets. Starting from the disease-gene associations of the ClinVar database (25), we found that the differential genes of aging were significantly associated with 12 diseases, and the differential genes of the immune system were significantly associated with 11 diseases (P-value ≤ 0.05). Interestingly, both are significantly associated with bowel cancer-related diseases, including colon carcinoma, hereditary nephrotic syndrome, and familial CRC (**Table 4**). These results indicated that aging and CD4+ T cell proportion may play important roles in the development of CRC.

**Table 4:** The significantly enriched ClinVar diseases of the aging- and CD4+ T cell proportion-derived differential genes.

Aging		CD4+ T cell proportion	
Disease	P-value	Disease	P-value
Hyperphosphatasia-intellectual disability syndrome	2.37e-03	Hyperphosphatasia-intellectual disability syndrome	5.05e-03
Diabetes mellitus type 2	3.68e-03	Severe congenital neutropenia	8.40e-03
Familial hyperinsulinism	9.11e-03	Neoplasm of stomach	1.28e-02
Neoplasm of stomach	9.11e-03	Familial colorectal cancer	2.31e-02
Congenital central hypoventilation	1.36e-02	Combined oxidative phosphorylation deficiency	3.39e-02
Carcinoma of colon	1.99e-02	Adult junctional epidermolysis bullosa	3.87e-02
Beckwith–Wiedemann syndrome	2.49e-02	Epidermolysis bullosa, junctional	3.87e-02
Hereditary nephrotic syndrome	2.49e-02	Familial adenomatous polyposis 1	3.87e-02
Hirschsprung disease	2.49e-02	Galloway–Mowat syndrome	3.87e-02
Combined oxidative phosphorylation deficiency	2.51e-02	Acute myeloid leukemia	4.02e-02
Neonatal diabetes mellitus	3.97e-02	Carcinoma of colon	4.60e-02
Familial colorectal cancer	4.17e-02		

### E3 Genes Associated With Aging

**Figure 2:**
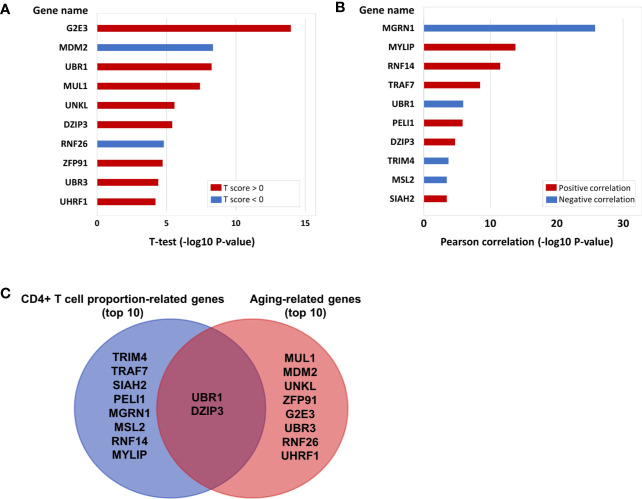
**(A)** The E3 gene list that is significantly associated with aging (top 10). **(B)** The E3 gene list that is significantly associated with CD4+ T cell proportion (top 10). **(C)** The intersecting genes that are significantly associated with both aging (top 10) and CD4+ T cell proportion (top 10).

**Figure 3:**
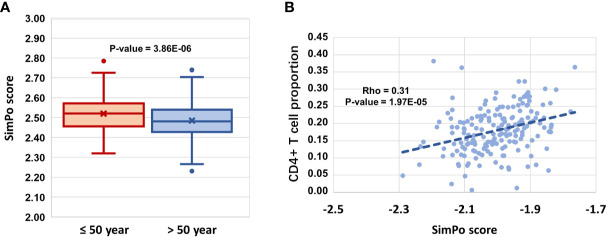
**(A)** *DZIP3* is significantly associated with aging. **(B)** *DZIP3* is significantly associated with the CD4+ T cell proportion.

In the above results, we not only proved that the aging immune system is related to the onset of CRC but also further verified that ubiquitinated genes play an important role in the aging immune system. Therefore, we conducted further analyses on the 233 E3 ubiquitin ligase genes. A total of 103 of the 233 E3 genes have cg probe annotations on the Illumina Infinium 450K Human Methylation Beadchip. Then, we obtained the SIMPO scores of 42 E3 genes. The methylation SIMPO scores of 23 E3 genes were significantly associated with the patient ages based on the adjusted T-test, and the E3 gene *DZIP3* ranked sixth (**Figure 2A** and **Figure 3A**) (**Table S3**).

### E3 Genes Associated With Immune Cell Proportions

Then, we obtained the SIMPO scores of 38 E3 genes based on the GSE69270 dataset. We calculated the Pearson correlations between the SIMPO scores of the 38 E3 ubiquitin ligases with immune cell proportions (including CD8+ T and CD4+ T cells, monocytes, granulocytes, and NK and B cells) and the sum of CD8+ T and CD4+ T cells. The Pearson correlation results showed that 15 E3 genes were significantly associated with the CD4+ T cell proportion (**Figure 2B** and **Table S4**), including the E3 gene *DZIP3* (**Figure 3B**). On the other hand, according to annotations provided by the GeneCards database (https://www.genecards.org/cgi-bin/carddisp.pl?gene=DZIP3), the DZIP3-encoded protein is significantly overexpressed in CD4+ T cells in the blood.

It is worth noting that there were two genes (*UBR1* and *DZIP3*) that appeared in the top ten genes related to aging and CD4+ T cell proportion (**Figure 2C**). Through literature searches in NCBI PubMed (https://www.ncbi.nlm.nih.gov/pubmed), it has been found that hundreds of published studies have demonstrated the associations of *UBR1* with cancers. Interestingly, however, no article has reported the association of *DZIP3* with colon cancer or even cancer. Considering the novelty of this gene, we conducted further clinical validation on *DZIP3*. In addition, according to the annotations in the Reactome pathway database (http://www.reactome.org/), *DZIP3* is involved in multiple immune-related pathways, including adaptive immune system, antigen processing- ubiquitination and proteasome degradation, class I MHC mediated antigen processing and presentation and immune system. Due to the strong association between immunity and cancer, we speculated that *DZIP3* may use the above immune-related pathways as the bridge to affect CRC.

### Survival Analysis Results

**Figure 4:**
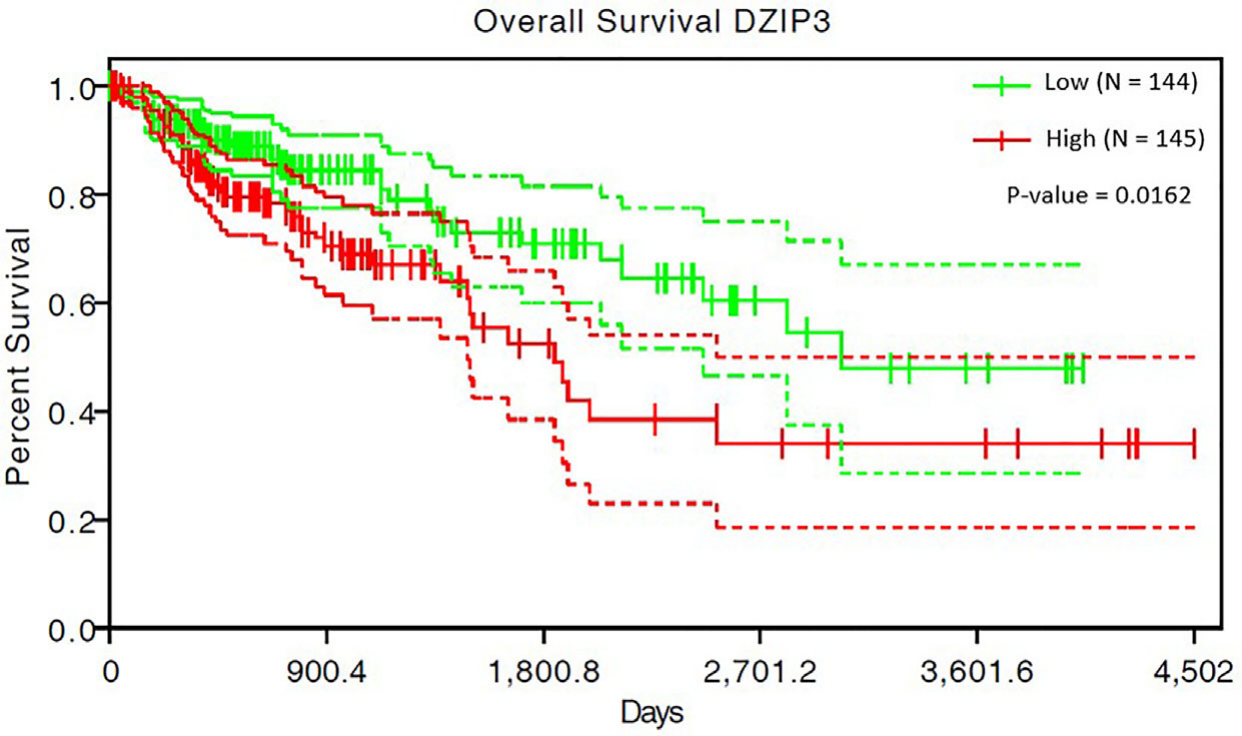
The survival analysis results of COAD patients for *DZIP3*.

The survival analysis of 293 COAD patients showed that the SIMPO scores of the three genes (*DZIP3*, *RNF26*, and *UBR3*) were significantly associated with the patient’s survival times. Among them, the most significant associated gene is *DZIP3*, which Hazard Ratio (HR) is 40.91 and P-value of Cox regression is 1.62e-02 (**Table 5** and **Figure 4**). This result demonstrated that compared to aging-related and CD4+ T cell proportion-derived gene *UBR1*, *DZIP3* may be more biologically related to CRC, which deserves further study.

**Table 5:** The survival analysis results of COAD patients for E3 genes.

Gene name	HR [exp(coef)]	coef	95% CI lower	95% CI upper	P-value
DZIP3	40.91	3.71	0.69	6.74	1.62e-02
RNF26	0.15	−1.89	−3.60	−0.19	2.95e-02
UBR3	0.17	−1.78	−3.46	−0.11	3.66e-02

### Methylation Variations in *DZIP3* Amplicon-cg14787155

**Figure 5:**
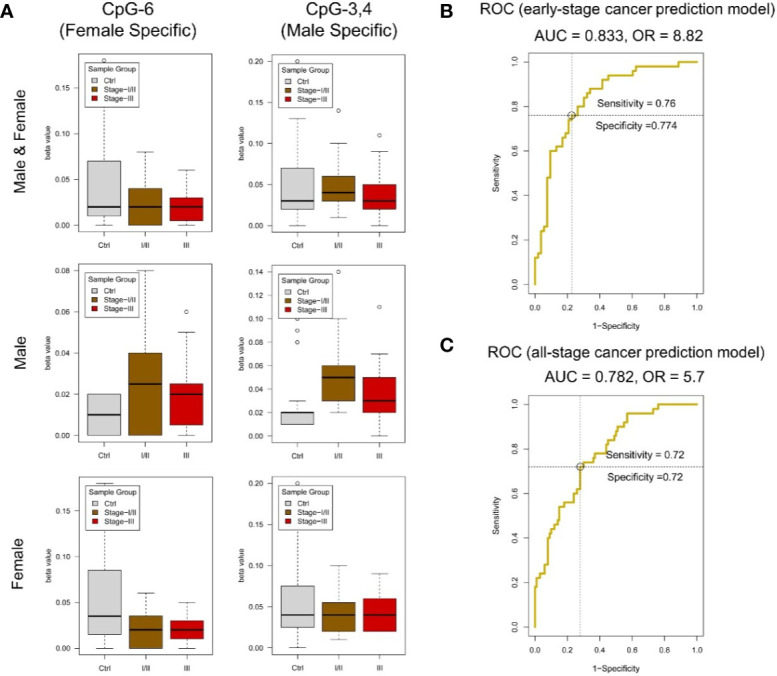
DNA methylation analysis of *amplicon-cg14787155* in *DZIP3*. **(A)** Sex-specific methylation level change between colorectal cancer and control in *amplicon-cg14787155*. **(B)** ROC analysis of the early-pTNM-stage colorectal cancer prediction model based on the MassARRAY data of *amplicon-cg14787155* features. The model with the best performance had an AUC of 0.833 and an OR of 8.82. **(C)** ROC analysis of the all-pTNM-stage colorectal cancer prediction model based on the MassARRAY data of *amplicon-cg14787155* features. The model with the best performance had an AUC of 0.782 and an OR of 5.7.

The clinicopathological characteristics of CRC patients are presented in **Table 6**. By MassARRAY analysis, we examined the methylation status of 25 CpG sites, and 15 methylation units were effectively detected in the majority of the samples for ***amplicon-cg14787155***. For each unit, we kept all the effective detected samples and tested the significance of methylation change in Ctrl *vs.* I and II and Ctrl *vs.* III. We found units showing significantly decreased methylation in CRC patients compared to normal controls (P-value ≤ 0.05, T-test). Specifically, we found that significantly changed methylation units were different between genders (**Figure 5A**, **Table S5**).

**Table 6:** Clinicopathological characteristics of the CRC patients (n = 150).

Variable	Patients (n = 100)	Control (n = 50)
Male/Female	67/33	18/32
Mean Age	62.07	65.74
Age range	27–68	16–86
TNM stage	I/II (53); III (47)	

### Colorectal Cancer Prediction Model Based on Amplicon-cg14787155

With EpiDesigner software, we obtained the sequence of the MassARRAY primer relative to the amplicon-cg14787155 region (**Table 7**). Based on the effective detected methylation data of 15 methylation units, we constructed a prediction model to classify CRC individuals from normal controls. As showed above, sex would be an important factor in predicting cancer state, and we also set sex as an input factor in the prediction model.

**Table 7:** Sequence of MassARRAY primer and position relative to ***amplicon-cg14787155***.

Amplicon	Primer	Sequence (5′->3′)
amplicon-cg14787155	10F	aggaagagagGGAAGTTTTTAGGTATTTTAGGGGAT
	T7R	cagtaatacgactcactatagggagaaggctTAAAACCCAAAATTCTTCTCCTCA

A data set of 150 samples (100 CRC in stage I/II/III and 50 normal controls) with effective detected values in all 15 methylation units was kept for the model construction. The data were analyzed in R (v3.6) environment, the caret (v6.0-84) and ROCR (v1.0-7) packages were used to construct the logistic regression model, which is essentially used to predict the probability of a binary (Cancer/Normal) event occurring. Logistic regression yields good performance to predict cancer outcome broadly in clinical cancer prediction studies. In this study, we collected the clinical CRC/normal sample information and chose the well-accepted logistic regression model to ensure the result could reflect the real clinical conditions. And we use leave-one-out cross-validation (LOOCV) to validate the model performance. First, we constructed the early-stage CRC prediction model in a dataset of 103 samples (53 CRC in pTNM stage I/II and 50 normal controls). In model construction, we set clinical “CRC/normal” outcomes as 0/1, gender information “male/female” as 1/0. We use gender information and all methylation levels of 15 effectively detected units in *amplicon-cg14787155* as inputs. R package *caret* was used to perform the model construction. We applied backward stepwise feature selection method, which begins with model containing all inputs and then iteratively removes the least useful input, one-at-a-time. Results showed that a model with the optimized input CpG sites (CpG_8, CpG_12, CpG_17, CpG_14.15, CpG_16, CpG_18, CpG_3.4, CpG_1.2, CpG_11, CpG_6) and sex showed the best performance (AUC = 0.833 and OR = 8.82) (**Figure 5B**).

Additionally, we constructed the all-stage CRC prediction model in the full data set. The model construction process was the same as early-stage CRC prediction model. A model with the optimized input CpG sites (CpG_12, CpG_18, CpG_1.2, CpG_6, CpG_11, CpG_3.4) and sex showed the best performance (AUC = 0.782 and OR = 5.7) (**Figure 5C**). The results showed that the methylation state of the *amplicon-cg14787155* region has specific features in predicting early-stage and all-stage CRC.

## Discussion

Ubiquitination is one of the main types of covalent modification of intracellular proteins. It participates in almost all life activities, such as cell survival, cell differentiation, and innate and acquired immunity (30–32). The abnormalities of ubiquitination are closely related to the occurrence of various tumors (33–37). It is worth noting that, consistent with the findings of this study, E3 ligase-mediated ubiquitination plays an important role in the development of CRC (38, 39). The in-depth study of ubiquitination will open up a new path for the study of CRC pathogenesis, diagnosis and treatment. The results show that ubiquitination is an important regulatory mechanism of the surface proteins of immune cells and plays an important regulatory role in aging and changes in the composition of immune cells. The next step is to focus on whether *DZIP3* plays an important role in the development of other types of cancer and on ubiquitinated pathway-modified proteins.

In this study, starting with E3 ubiquitin ligase genes, we screened early markers of CRC related to immune system aging and immune escape. The results showed that the ubiquitination regulatory gene *DZIP3* was significantly correlated with age and changes in immune cell components. In addition, previous studies have shown that genes originating in different stages have different biological functions, which are closely related to human phenotypes (40–45). Therefore, the evolutionary information of genes often implies important clues to the analysis of human disease mechanisms (40, 41, 44). For example, a study had shown that the gene network composed of eumetazoa-originated genes can regulate the hallmarks of cancer (44), reflecting the potential associations of eumetazoa-originated genes with cancer. Interestingly, through Liebeskind’s research on the origin of human genes (46), *DZIP3* originated in the eumetazoa stage, suggesting that *DZIP3* may have an influence in the progression of cancer to a certain extent. Furthermore, the survival analysis for 293 COAD patients showed that DNA methylation value of *DZIP3* is significantly associated with the patient’s survival times. Considering the association between *DZIP3* and cancer, we designed a low-cost nucleic acid mass spectrometry kit, which was validated by recruiting patients with early CRC.

At present, the US FDA-approved CRC screening methods mainly include colonoscopy, fecal DNA testing, the fecal occult blood test (FOBT), the conventional blood carcinoembryonic antigen (CEA) indicator, and the circulating methylated SEPT9 test. However, the above technologies still have certain limitations. For example, the early preparation of colonoscopy is cumbersome, invasive and subject to the subjective influence of the operator’s skills and clinical experience. Based on circulating methylated SEPT9 DNA for detecting CRC in a study by Church et al., the methylation of SEPT9 DNA had a sensitivity of 35.0% for patients with stage I CRC, 63% for stage II, 46.0% for stage III, and 77.4% for stage IV, with a specificity of 91.5% (47). In recent years, peripheral blood circulating tumor cells (CTCs) have made great progress, and this technique is convenient for blood collection, with high public acceptance. However, there are still many problems in the application of CTCs. For example, positive results of CTCs still require confirmation by colonoscopy, and the appropriate testing interval of CTCs should be further explored.

However, this study used the DNA methylation characteristics of *DZIP3* as early screening markers of early-stage CRC (sensitivity = 0.760, specificity = 0.774) and all-stage CRC (sensitivity = 0.720, specificity = 0.720). In addition, this screening technique only needs to extract the whole blood of individuals for MassARRAY analysis; the samples are easy to obtain, and the results are objective. Therefore, this method may have important applications in clinical CRC screening. we concluded that DZIP3 has the potential to become an effective biomarker of CRC, which is worthy of experimental verification *in vivo* and *in vitro* in the future.

Another interesting feature of the present study suggests a novel early cancer screening approach based on blood cells. This blood cell methylation analysis mainly reflects the status of the immune system rather than cancer cells. Traditionally, liquid biopsy is focused on DNA mutation or DNA methylation tests on circulating free DNAs, which are mainly released by cancerous cells. Our results confirmed that aging and immune escape are important links in the development of intestinal cancer. Considering that a certain frequency of gene mutations will occur in healthy people and healthy tissues, the speed-limiting step of cancer occurrence, that is, the bottleneck, is not gene mutation but changes in the immune system. As cancer cells and immune systems reflect the yin-yang aspects of tumorigenesis, our results raise a new option for detecting the early and rate-limiting steps of tumorigenesis. Considering that blood cell methylation analysis could be conducted in healthy individuals, it is possible that our method could outline a continuous curve of healthy, inflammation and transformation processes from enteritis to cancer. Thus, our method holds specific value for use in health management compared to the traditional liquid biopsy method, in which strong signals will appear only after enough cancer cells have been produced.

## Pre-print statement

We have made a pre-print version of this manuscript available on the bioRxiv (The bioRxiv 2020.01.13.905299; doi: https://doi.org/10.1101/2020.01.13.905299).

## Data Availability Statement

Publicly available datasets were analyzed in this study, these can be found in the NCBI Gene Expression Omnibus (GSE40279 and GSE69270). The raw data supporting the conclusions of this article will be made available by the authors, without undue reservation.

## Ethics Statement

The studies involving human participants were reviewed and approved by the institutional ethics committee of the Guangdong Provincial People’s Hospital and FoShan New RongQi Hospital (No.GDREC2016203H(R1)). The patients/participants provided their written informed consent to participate in this study.

## Author Contributions

YQ and FL conducted the data mining and bioinformatics analyses. DW, XY, ZL, and QY performed the clinical sample and data collection. YL and ZN were responsible for the clinical trial design and organization. ZN and LL performed part of the clinical data mining. YZ, YC, and AW took part in the lab experimental design. ZH, DT, and BH took part in designing the clinical validation. RX helped in preparing the manuscript. JX led the epigenetic research and designed the strategy for the integrated analysis of DNA methylation data. YQ, FL, and JX wrote the manuscript. All authors contributed to the article and approved the submitted version.

## Funding

This research was partly funded by grants from the Shenzhen Science & Technology Program (CKFW2016082915204709, JCYJ20151029154245758, CYZZ20160530183500723) and the Science and Technology Planning Project of Guangdong Province, China (No. 2016A020215128).

## Conflict of Interest

ZN and LL were employed by the company Shenzhen Taikontek Health technology Co., Ltd.

The remaining authors declare that the research was conducted in the absence of any commercial or financial relationships that could be construed as a potential conflict of interest.
